# Ein 27-jähriger Mann mit „Hornhauttrübung“ nach Astverletzung

**DOI:** 10.1007/s00347-020-01130-5

**Published:** 2020-06-03

**Authors:** Loïc Hamon, Elias Flockerzi, Navid Ardjomand, Berthold Seitz, Loay Daas

**Affiliations:** 1 Klinik für Augenheilkunde, Universitätsklinikum des Saarlandes (UKS), Kirrbergerstr. 100, Gebäude 22, 66421 Homburg/Saar, Deutschland; 2 Sehzentrum für Augenlaser und Augenchirurgie, Graz, Österreich

## Anamnese

Ein 27-jähriger Patient stellte sich zum ersten Mal in unserer Sprechstunde zur Mitbeurteilung bei scharf begrenzter „Hornhauttrübung“ am linken Auge vor. Der Patient gab an, dass er vor ca. 20 Tagen beim Wandern einen Baumast in das linke Auge bekommen habe. Seitdem bemerke er eine zunehmende Visusminderung am betroffenen linken Auge. Bei persistierenden Beschwerden konsultierte er seinen Augenarzt, der ihn bei anscheinender Inaktivität der „Trübung“ zur Mitbeurteilung überwies.

Am Tag der Vorstellung berichtete der Patient sowohl über eine persistierende und zunehmende Visusminderung als auch Photophobie am betroffenen linken Auge. Er litt unter keinen Augenschmerzen. Der Patient gab an, dass bei ihm 2016 bei mäßiger Hyperopie (+3 dpt) eine laserassistierte In-situ-Keratomileusis (LASIK) mit Mikrokeratom beidseits durchgeführt wurde. Er gab sonst keine Augen- oder allgemeine Vorerkrankungen an und nimmt keine systemische Medikation ein.

## Klinischer Befund

**Figure 1:**
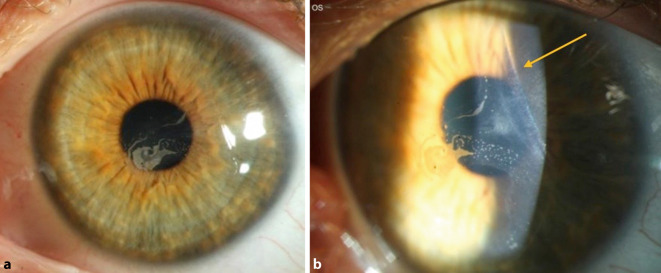

Der bestkorrigierte Visus betrug bei Erstvorstellung 0,4 am betroffenen linken Auge (subjektive Refraktion: +2,00/−1,50/A 175°) sowie 0,8 am rechten Partnerauge (subjektive Refraktion: +1,75/−1,00/A 9°). Spaltlampenbiomikroskopisch zeigte sich rechts ein regelrechter Befund und ein LASIK-Flap mit klarem Interface. Links zeigten sich eine inselartige Ansammlung von Zellen unter dem LASIK-Flap vom nasalen Flaprand bis zur optischen Achse sowie eine parazentrale Lentikelfalte des LASIK-Flaps (Abb. 1). Der Flap-Hinge war nasal anliegend, die Vorderkammer tief und reizfrei, die Iris intakt und die Linse klar. Fundoskopisch zeigte sich ein regelrechter altersentsprechender Befund beidseits.

## Diagnostik

**Figure 2:**
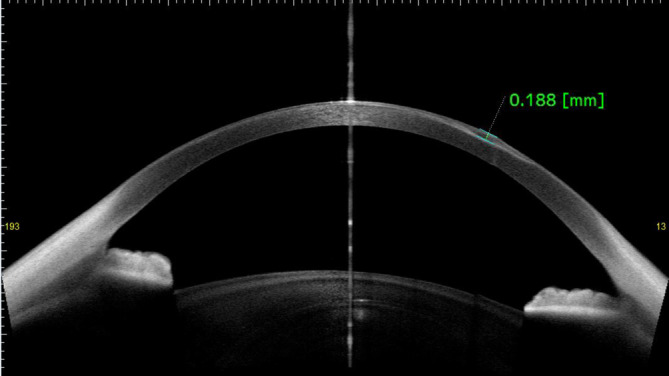

In der optischen Kohärenztomographie des vorderen Augenabschnitts (VA-OCT) beobachteten wir am linken Auge bei ca. 180 µm Tiefe eine hyperreflektive, gut definierte Struktur (Abb. 2).

## Wie lautet Ihre Diagnose?

## Definition

Die Epithelinvasion ist eine seltene postoperative Komplikation bei Zustand nach LASIK. Diese wird in der Literatur zwischen 0 und 3,9 % der Patienten postoperativ angegeben [2, 6]. Die Pathogenese ist umstritten. Die beiden akzeptierten Hypothesen sind eine intraoperative mechanische Übertragung von Epithelzellen oder eine postoperative Invasion vom Rand des LASIK-Flaps [7]. Während zahlreiche Risikofaktoren für spontane Epithelinvasion beschrieben sind (u. a. Hyperopiekorrektur, Alter des Patienten, Diabetes mellitus Typ 1, Zustand nach operativer Revision wie „flap repositioning“ oder „flap lifting“) [6], bleibt daneben die posttraumatische Epithelinvasion unter dem LASIK-Flap als häufige Ursache.

Diagnose**:** Posttraumatische LASIK-Flap-Einfaltung mit Epithelinvasion (Grad 4 nach Probst/Machat)

Die Epithelinvasion wurde 1996 von Probst und Machat in 4 Grade klassifiziert [4] und vor Kurzem von Neff und Probst revidiert [3]: Grad 1: dünn, nicht progressiv, nahe am Flaprand, ohne Progression und ohne Therapiebedarf; Grad 2: dünn, langsam progressiv, nahe am Flaprand, mit nicht dringendem Bedarf einer operativen Therapie; Grad 3 und Grad 4: dicke, undurchsichtige, schnell progressive Epithelinvasion, mehr als 2 mm vom Flaprand entfernt für Grad 3 oder die optische Achse betreffend für Grad 4. Die Grade 3 und 4 benötigen eine dringende operative Versorgung. Differenzialdiagnostisch sollte eine diffuse lamelläre Keratitis (DLK) (sog. „Sand of Sahara“ [SOS]) ausgeschlossen werden. Diese zeigt sich spaltlampenbiomikroskopisch als Infiltrat unter dem LASIK-Flap mit unterschiedlicher Ausprägung und Ausdehnung je nach Stadium. Stadieneinteilung und Therapie wurden von Bühren et al. genau beschrieben [1].

## Therapie und Verlauf

**Figure 3:**
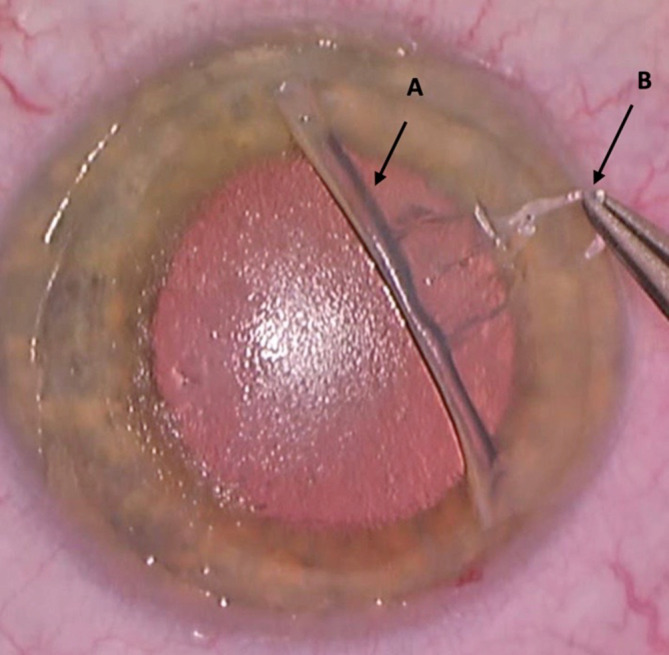

**Figure 4:**
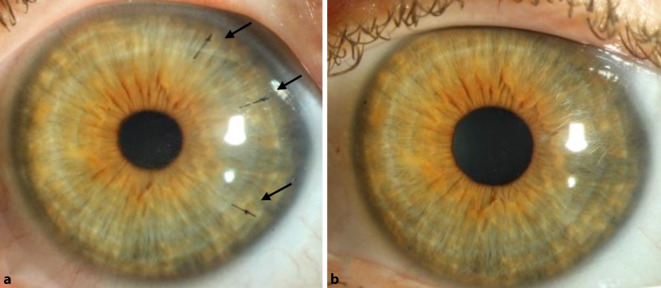

**Figure 5:**
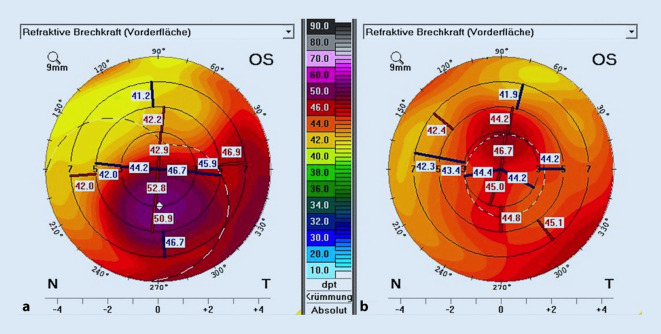

Wir leiteten am Tag der Diagnose die Therapie mit Prednisolonacetat 10 mg/ml Augentropfen (AT) stündlich ein und planten für den nächsten Tag eine LASIK-Flapanhebung mit mechanischer Exzision der epithelialen Zellen vom stromalen Bett sowie von der Rückfläche des Flaps (Abb. 3), Flapspülung (mit „balanced salt solution“ [BSS], anschließend mit konservierungsfreien Antibiotika), Applikation von 0,02 % Mitomycin C (MMC) als Schwämmchen auf Bett und Flaprückseite während 60 s und Anlegen von 3 Einzelknüpfnähten an den zurückgeklappten Flapränden (für 4 Wochen [Abb. 4a]). Die Kombination von mechanischer Abtragung und Applikation von MMC wurde von Wilde et al. als sicher, mit geringer Rezidivrate und mit einer guten Visusrehabilitation dargestellt [8]. Bei nicht ausreichender mechanischer Abtragung des Epithels mittels Instrumenten bleibt die Option einer zusätzlichen Excimerlaser-phototherapeutischen Keratektomie (Excimer-PTK) von Bett und Flaprückfläche [5]. Als lokale Therapie bekam der Patient postoperativ Moxifloxacin AT 5‑mal/Tag für 1 Woche und Prednisolonacetat 10 mg/ml AT 5‑mal/Tag für 4 Wochen. Bei der letzten postoperativen Kontrolle – 4 Monaten nach der Operation – zeigte sich ein regelrechter und komplikationsloser Befund ohne Hinweis auf Rezidiv (Abb. 4b). Der Visus betrug 1,0 am betroffenen Auge (sine correctione). Topografisch zeigte sich eine deutliche Regularisierung nach dem Eingriff (Abb. 5). Die weiteren Kontrollen wurden durch den niedergelassenen Augenarzt durchgeführt.

## Fazit für die Praxis

- Eine ausführliche Anamnese spielt bei unklarem Hornhautbefund eine entscheidende Rolle für die Diagnosestellung und Therapie.
- Der Begriff „Hornhauttrübung“ sollte als Befund oder gar Diagnose vermieden werden.
- Das VA-OCT kann helfen, die Tiefe der Pathologie exakt zu bezeichnen.
- Bei progressiver oder die optische Achse betreffender Epithelinvasion ist eine dringende operative Intervention, unabhängig vom Grad der Visusminderung, indiziert.
- Die mechanische Entfernung des Epithels von Bett und Flaprückfläche in Kombination mit 0,02 % Mitomycin C für 60 s scheint derzeit die Methode erster Wahl zu sein.
- Eine intraoperative Mydriasis kann dem Operateur helfen, durch den Rotreflex des Fundus auch geringste Spuren von Epithel zu identifizieren und zu entfernen.
